# New Record for the Coffee Berry Borer, *Hypothenemus hampei*, in Hawaii

**DOI:** 10.1673/031.011.11701

**Published:** 2011-09-12

**Authors:** Elsie Burbano, Mark Wright, Donald E. Bright, Fernando E. Vega

**Affiliations:** ^1^ University of Hawaii, Department of Plant and Environmental Protection Sciences, College of Tropical Agriculture and Human Resources, 3050 Maile Way, Honolulu, HI 96822; ^2^ Colorado State University, Department of Bioagriculture Sciences and Pest Management, Fort Collins, CO 80523; ^3^ Sustainable Perennial Crops Laboratory, U. S. Department of Agriculture, Agricultural Research Service, Bldg 001, BARC West, Beltsville, MD 20705

**Keywords:** bark beetle, broca, Scolytinae

## Abstract

The coffee berry borer, *Hypothenemus hampei* (Ferrari) (Coleoptera: Curculionidae) is endemic to Africa and is the most devastating pest of coffee worldwide. The female bores a hole in the coffee berry and deposits her eggs inside. Upon hatching, larvae feed on the seeds, thus reducing both quality and yields of the marketable product. The coffee berry borer was found in the district of Kona on the island of Hawaii in August 2010 and appears to be restricted to that area.

## Introduction

**Figure 1.**
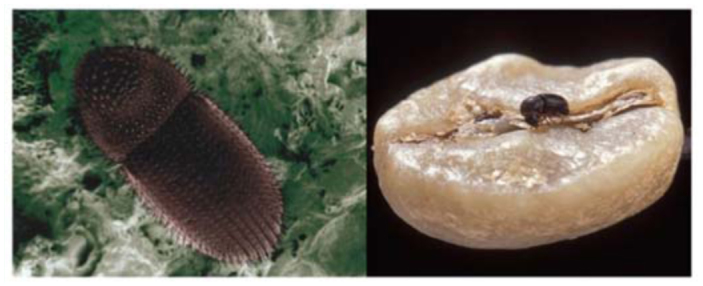
Dorsal view of an adult coffee berry borer, *Hypothenemus hampei* (left) and adult female (∼ 2 mm long) walking over a coffee seed (right). Photos by Eric Erbe, USDA, ARS (left) and Peggy Greb, USDA, ARS (right). High quality figures are available online.

**Figure 2.**
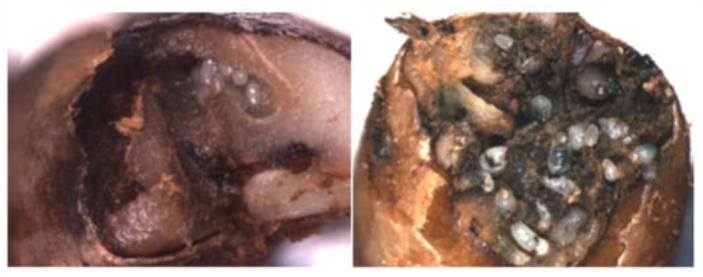
*Hypothenemus hampei* galleries containing eggs (left), and eggs and larvae (right). Photos by Elsie Burbano. High quality figures are available online.

The coffee berry borer, *Hypothenemus hampei* (Ferrari) (Coleoptera: Curculionidae, Scolytinae; Figure 1), is a bark beetle endemic to Central Africa and now distributed throughout all coffee producing countries in the world, with the exception of Nepal and Papua New Guinea. It is the most economically important coffee pest worldwide (Le Pelley 1968; Vega 2008). Colonizing females bore a hole in the coffee berry and deposit eggs within galleries, followed by larval feeding on the coffee seed (Figure 2). This reduces both yield and quality of coffee, which in turn affects the income of coffee growers (Damon 2000; Jaramillo et al. 2006). There is sibling mating among the adult progeny with a 10:1 sex ratio favoring females. Therefore, when new adult females emerge from the berry, they are already inseminated and ready to locate another berry in which to continue their life cycle (Vega et al. 2009). Male insects do not fly and remain inside the berry.

## Discovery of *H. hampei* in Kona

**Figure 3.**
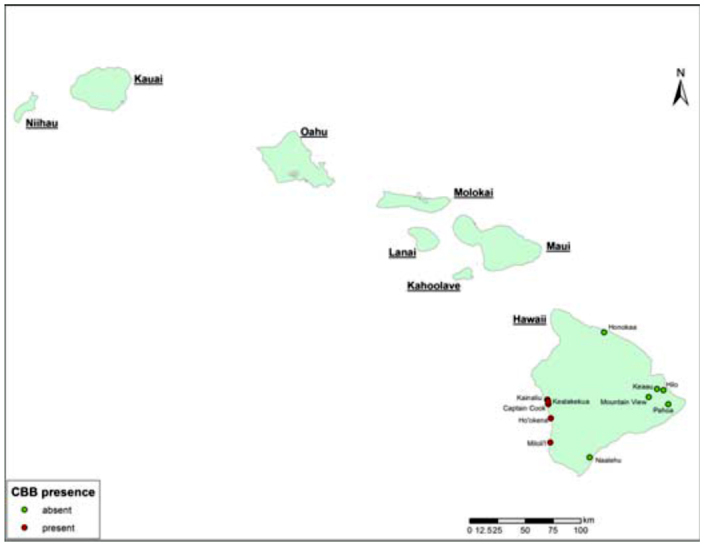
Distribution of *Hypothenemus hampei* in the district of Kona, Island of Hawai'i. Pest incidence data by Hawaii Department of Agriculture. Map courtesy of Kai Sonder, CIMMYT. High quality figures are available online.

In August 2010, *H. hampei* was found in South Kona, Island of Hawaii. Specimens were identified by Donald E. Bright of Colorado State University, Al Samuelson of Bishop Museum, and Natalia J. Vandenberg of the Systematic Entomology Laboratory, USDA-ARS. The infestation in South Kona extends from Kainaliu to Opihihale (Figure 3; Hawaii Department of Agriculture 2010a), which indicates the insect has been present on the island for some time. The insect has not yet been found on any other Hawaiian island.

Among the usual pest management options that can be implemented against *H. hampei* are the use of biological control agents, such as parasitoids and fungal entomopathogens such as *Beauveria bassiana.* One promising area is the identification of attractants and/or repellents that can be used against the insect. These pest management options were recently reviewed by Vega et al. (2009). In addition, crop sanitation could hold promise in reducing pest impact. This strategy would require an agreement among growers to harvest and destroy infested berries (Bustillo et al. 1998). Removing the food source for *H*. *hampei* could have a significant impact while the beetle still has a very limited distribution across Hawaii. As of 30 September 2010, only 8 farms were known to be infested in Kona (Department of Agriculture 2010b).
